# 

**Published:** 1979

**Authors:** 


### Contents

3 The Evolution of Medical Societies in Britain — Have They a Future?
Donald S. Ractliffe

Obituary

11 W. G. J. Hampson, M.A., B.M., B.Ch., F.R.C.S.Ed.

12 Dr. Clifford D. Evans

